# Metformin for Longevity and Sarcopenia: A Therapeutic Paradox in Aging

**DOI:** 10.3390/biomedicines14020376

**Published:** 2026-02-05

**Authors:** Song-Yi Han, Mukesh Kumar Yadav, Jing-Hua Wang

**Affiliations:** 1 College of Korean Medicine, Dongguk University, 32 Dongguk-ro, Goyang 10326, Gyeonggi-do, Republic of Korea; syh12156@gmail.com; 2 Department of Microbiology, Central University of Punjab, Bathinda 151401, Punjab, India; mukesh.yadav@cup.edu.in; 3 Institute of Oriental Medicine, Dongguk University, 32 Dongguk-ro, Goyang 10326, Gyeonggi-do, Republic of Korea

**Keywords:** metformin, sarcopenia, aging, AMPK, mTORC1, skeletal muscle, resistance training, frailty

## Abstract

Metformin is a first-line oral antidiabetic agent that has attracted increasing interest as a potential geroprotective therapy due to its ability to improve metabolic homeostasis, reduce oxidative stress, and attenuate chronic inflammation. However, its role in skeletal muscle aging and sarcopenia remains controversial. Observational and epidemiological studies suggest that metformin use is associated with a lower prevalence of sarcopenia, particularly in metabolically compromised or insulin-resistant older populations, where improvements in systemic metabolism and inflammatory burden may indirectly support muscle quality and function. In contrast, randomized interventional trials in metabolically healthy older adults indicate that metformin can blunt resistance exercise–induced muscle hypertrophy and protein synthesis, likely through sustained activation of AMP-activated protein kinase (AMPK) and consequent suppression of mammalian target of rapamycin complex 1 (mTORC1) signaling. This perspective argues that these apparently opposing outcomes reflect a con-text-dependent therapeutic paradox rather than inconsistent evidence. Metformin may provide metabolic protection in frail, insulin-resistant individuals, yet limit anabolic adaptations in physically active older adults. These findings emphasize the necessity for precision geropharmacological strategies to balance metabolic longevity with preservation of musculoskeletal health in aging populations.

## 1. Introduction

Initially prescribed for type 2 diabetes mellitus (T2DM), metformin has recently gained significant attention as a potential geroprotective agent [1]. It produces multiple effects by inhibiting mitochondrial complex I, decreasing hepatic gluconeogenesis, activating AMP-activated protein kinase (AMPK), and influencing inflammatory and oxidative stress pathways [2,3,4]. The Targeting Aging with Metformin (TAME) trial and related studies have expanded its reputation beyond glycemic control to possible anti-aging uses [5]. However, skeletal muscle (the largest metabolic organ) complicates this issue. Age-related muscle loss, or sarcopenia, not only leads to frailty and disability but also worsens metabolic disorders [6]. Intriguingly, while large observational studies suggest protective effects, intervention trials in older adults show that metformin may inhibit muscle gains from resistance training [7,8]. These seemingly contradictory findings suggest that the effects of metformin on aging are not uniform but instead vary systematically across disease environments and experimental conditions.

As shown in Table 1, a representative overview of selected recent studies illustrates the heterogeneous effects of metformin across aging-related conditions. Meta-analyses and clinical studies report inconsistent or modest benefits in neurodegenerative and cognitive disorders, with several investigations indicating no overall protective effect or even potentially adverse associations in specific subpopulations [9,10,11,12]. In addition, preclinical models demonstrate relatively consistent beneficial effects, including reduced tau pathology, enhanced autophagy, and improved cellular stress resistance, largely mediated through AMPK activation, mTORC1 suppression, and anti-inflammatory pathways [13,14,15]. Notably, these effects vary according to disease stage, age, experimental model, and dosage, underscoring the absence of a uniform therapeutic profile. Accordingly, this heterogeneity indicates that metformin may not represent a “universal” solution for extending lifespan.

## 2. The Paradox: Restrained Muscle Adaptation and the AMPK-mTOR Signaling Dilemma

In a randomized controlled trial (the MASTERS study), older adults who received 1700 mg/day of metformin during 14 weeks of progressive resistance training gained significantly less muscle mass and cross-sectional area compared to the placebo group [8]. Transcriptomic profiling further revealed attenuated activation of anabolic and mitochondrial biogenesis pathways in the metformin group [19]. Mechanistically, this paradox can be partially explained by dysregulated AMPK–mTORC1 crosstalk [20]. Metformin activates AMPK, which, while beneficial for metabolic regulation, concurrently inhibits mTORC1, the central driver of muscle protein synthesis [21,22]. In healthy muscle, this may blunt hypertrophic adaptation to mechanical loading. Therefore, the same signaling pathway that provides metabolic protection may impair muscle growth when anabolic stimulation is required.

Furthermore, in vitro studies confirm this concern. Metformin exposure in C2C12 myotubes increases FoxO3a expression and nuclear localization and enhances its binding to the myostatin promoter, thereby activating a FoxO3a-dependent catabolic program that is classically linked to induction of the muscle atrophy-related E3 ubiquitin ligases MuRF1 and Atrogin-1 [23,24]. These molecular patterns are consistent with disuse or fasting-induced atrophy, raising the possibility that chronic metformin use under specific conditions could mimic aspects of catabolic stress [25]. Even more patently, in clinical trials combining metformin with resistance exercise, older subjects exhibited reduced gains in strength and muscle hypertrophy, despite preserved insulin sensitivity [8,26]. This dissociation is consistent with what may be conceptualized as a ‘geroprotection paradox’, in which cellular signaling is improved while tissue-level adaptation is weakened.

In support of this concept, the mechanistic evidence summarized in Table 2 shows the strongly context-dependent and often bidirectional effects of metformin on skeletal muscle aging. Across in vivo, in vitro, and limited clinical observations, metformin is frequently associated with partial improvements in muscle quality, cellular stress resistance, and metabolic maintenance, such as enhanced autophagy, mitochondrial function, and reduced inflammatory burden, particularly in aging or metabolically compromised models, largely through AMPK activation and related pathways [27,28,29]. However, these cellular benefits are not consistently accompanied by enhanced muscle mass or anabolic capacity, as metformin exposure in several settings is linked to attenuated myogenic signaling, altered satellite cell–related markers, activation of catabolic transcriptional programs, or negligible effects on fat-free mass in randomized trials [30,31,32]. Importantly, these outcomes vary substantially with experimental model, age, metabolic status, treatment duration, and dosage, emphasizing the absence of a uniform therapeutic profile and providing putative mechanistic support for the dissociation between improved cellular homeostasis and blunted hypertrophic adaptation described above.

Beyond signaling mechanisms, population characteristics further shape the apparent paradox observed across study designs. The apparent discrepancy between observational and interventional findings can be largely attributed to differences in the populations studied and the outcomes assessed. Observational studies predominantly involve older adults with metabolic comorbidities, in whom sarcopenia develops in parallel with systemic metabolic stress and inflammation; in this context, metformin may indirectly preserve muscle quality and reduce sarcopenia risk through its metabolic and anti-inflammatory effects. By contrast, interventional trials typically enroll metabolically healthy, physically active older adults undergoing resistance training, where muscle adaptation relies on intact anabolic signaling and transient mTORC1 activation. In this setting, chronic AMPK activation by metformin may attenuate mTORC1-driven protein synthesis and blunt hypertrophic responses despite improved metabolic indices. Taken together, these findings are not contradictory but complementary, reflecting fundamental differences in biological context and study design. This perspective sets the stage for a broader discussion of context-dependent modifiers, including nutrition, exercise modality, and the gut microbiome.

## 3. Understanding Context-Dependent Metformin Effects in Aging

One key reason for the inconsistent findings across studies in Table 2 is that the effects of metformin strongly depend on context. Its influence differs with age, metabolic health, nutritional status, physical activity level, and gut microbiota. Together, these factors influence the balance between muscle-building and muscle-breaking pathways in aging muscle, which ultimately determines whether metformin is beneficial or harmful.

In metabolically unhealthy or diabetic individuals, insulin-sensitizing and anti-inflammatory properties of metformin may partially restore impaired anabolic responsiveness [38]. By improving systemic glucose handling, reducing low-grade inflammation, and alleviating lipid-induced metabolic stress, metformin may indirectly enhance muscle quality, even in the absence of robust gains in muscle mass [39]. Such effects are consistent with observations in sarcopenic or metabolically compromised models, where improvements in muscle function and cellular integrity often outweigh changes in absolute muscle quantity [36]. In contrast, in metabolically healthy or physically trained older adults, chronic activation of AMPK by metformin may blunt the mTORC1 signaling required for resistance exercise-induced muscle hypertrophy [8]. Because adaptive muscle growth relies on transient mTORC1 activation, sustained AMPK dominance may attenuate training responsiveness, providing a mechanistic basis for reports of diminished hypertrophic adaptations in this population. Interpretation is further complicated by sex-specific and dose-dependent factors. Age-related changes in pharmacokinetics, renal clearance, and muscle perfusion may alter tissue exposure to metformin, while hormonal differences between males and females could modulate downstream signaling responses [40,41,42]. These variables are rarely harmonized across studies, contributing additional layers of heterogeneity.

Nutritional status also represents a central determinant of this balance. Adequate protein intake is required to elicit postprandial mTORC1 activation and sustain muscle protein synthesis in older adults, who already exhibit age-related anabolic resistance [43]. Therefore, under conditions of insufficient protein or energy intake, metformin-mediated suppression of basal mTORC1 tone may further inhibit anabolic signaling capacity, thereby predisposing aging muscle to protein loss.

Moreover, the interaction between metformin and exercise is also critical. Aerobic exercise, which relies primarily on mitochondrial oxidative metabolism, may act synergistically with the mitochondrial and redox-modulating effects of metformin [44]. In contrast, resistance training relies on short-lived mTOR activation for muscle remodeling, a process that metformin can suppress [45]. However, muscle-intrinsic signaling pathways alone may not fully account for the divergent muscular outcomes observed across populations [46,47].

Metformin is well known to remodel gut microbial composition and function, including enrichment of short-chain fatty acid (SCFA)–producing taxa and alterations in bile acid metabolism, both of which can influence host energy homeostasis, inflammation, and muscle metabolism [46]. Exercise itself is a potent modulator of the gut microbiome, and the combined effects of metformin and specific exercise modalities may generate distinct microbial and metabolite profiles that differentially impact muscle anabolic signaling [47,48]. Although these interactions remain incompletely characterized, they introduce an additional layer of biological variability that may help explain population-specific responses to metformin.

Building on these observations, the gut microbiome can be further conceptualized as a signaling intermediary linking metformin exposure to skeletal muscle adaptation. Metformin-induced shifts in microbial composition, including increased SCFA production and remodeling of bile acid–transforming communities. These changes may influence host metabolism through elevated circulating SCFAs and modified bile acid pools, which regulate systemic inflammation, insulin sensitivity, and energy homeostasis. From a muscle signaling perspective, SCFAs such as butyrate and propionate may indirectly modulate skeletal muscle anabolic responsiveness by attenuating chronic low-grade inflammation and improving insulin signaling, thereby lowering basal metabolic stress in aging muscle. In parallel, and through partially overlapping mechanisms, microbiota-driven alterations in bile acid composition may influence muscle metabolism via FXR- and TGR5-dependent pathways, with downstream effects on mitochondrial function, oxidative capacity, and AMPK activity. Collectively, these microbiome-derived signals may shift the balance between AMPK-mediated metabolic stress responses and mTORC1-dependent anabolic signaling in skeletal muscle.

Importantly, this framework does not propose a deterministic causal pathway, but rather a hypothesis-driven model in which gut microbiota composition acts as a contextual modifier of metformin’s muscular effects. Depending on baseline metabolic status, exercise context, and microbial configuration, metformin-induced microbiome remodeling may either support muscle maintenance in metabolically compromised individuals or fail to compensate for AMPK-driven suppression of hypertrophic signaling in physically active older adults. As such, this model highlights the gut–muscle axis as a testable mechanistic layer within precision geropharmacology and underscores the need for future studies integrating microbiome profiling with muscle signaling and functional endpoints.

Overall, these observations argue strongly against a “one-size-fits-all” strategy for metformin use in sarcopenia and highlight the necessity of precision-based approaches that consider metabolic status, nutritional adequacy, exercise type, age, and sex. The growing recognition of context-dependent responses to metformin highlights the need for future precision geropharmacological approaches supported by actionable biomarkers. Potential candidates include baseline AMPK-mTORC1 signaling activity, systemic inflammatory markers (e.g., CRP and IL-6), metabolic indices of insulin resistance, and emerging gut microbiome-derived signatures. These biomarkers may enable stratification of individuals who derive metabolic benefit from metformin versus those at risk of impaired anabolic adaptation, and should be evaluated in future studies.

## 4. Redefining Geroprotection in Muscle Aging

The binary view of metformin should be abandoned as either a “beneficial” or “harmful” drug for skeletal muscle. Instead, a nuanced framework is needed that integrates dose–response relationships, timing of administration, and combined lifestyle interventions. Emerging evidence suggests that intermittent or lower-dose metformin may mitigate the hypertrophy-blunting effect while preserving mitochondrial and anti-inflammatory advantages [49]. Alternatively, co-treatment strategies that transiently relieve AMPK-mediated suppression could achieve balanced outcomes, such as metformin cycling or combination with nutraceutical AMPK modulators [50,51].

Moreover, hybrid compounds like RJx-01 (metformin + galantamine) have demonstrated synergistic effects in preclinical models, improving autophagy, neuromuscular junction integrity, and muscle force generation beyond what either compound achieves alone [27]. Such findings indicate that polypharmacological approaches may overcome metformin’s intrinsic limitations.

Importantly, future research must differentiate between “muscle aging” (cellular senescence, mitochondrial decline, autophagic failure) and “sarcopenia” (loss of mass and function). While these phenomena overlap, metformin may target the former more effectively than the latter. Longitudinal human trials that integrate muscle omics, imaging, and functional endpoints are urgently needed to determine whether metformin delays true sarcopenia or merely improves metabolic biomarkers.

One promising strategy to reconcile the metabolic benefits of metformin with its potential to blunt anabolic adaptation is the exploration of intermittent or cycling dosing paradigms. From a mechanistic perspective, the beneficial effects of metformin on mitochondrial function, inflammation, and metabolic homeostasis are largely mediated through AMPK activation and downstream stress-response pathways, which may not require continuous pharmacological pressure to be sustained. In contrast, chronic AMPK dominance may interfere with the transient mTORC1 activation required for resistance exercise–induced muscle protein synthesis. Intermittent exposure to metformin could therefore represent a theoretical means to retain metabolic protection while permitting periodic restoration of anabolic responsiveness during phases of mechanical loading or nutritional sufficiency. In addition, co-treatment strategies warrant consideration as a conceptual way to balance metformin’s pleiotropic effects. Rather than opposing metformin’s action directly, rational combinations may aim to temporally or contextually offset its anabolic suppression. For example, pairing metformin with nutritional, exercise-based, or pharmacological interventions that support mTORC1 signaling, satellite cell activity, or neuromuscular adaptation could help preserve muscle adaptability without negating metabolic benefits. Importantly, these approaches should not be viewed as fixed therapeutic prescriptions, but as testable hypotheses that underscore the necessity of context-aware intervention design. Within a precision geropharmacology framework, such strategies emphasize timing, physiological state, and adaptive capacity as critical determinants of outcome, reinforcing the view that metformin’s effects on aging muscle are modifiable rather than intrinsically paradoxical.

## 5. Re-Evaluating the “Anti-Aging” Potential of Metformin

Interest in metformin as a potential universal anti-aging drug has sometimes exceeded the strength of the available evidence. Its use in large longevity trials, such as the TAME study [5], highlights the need to evaluate not only effects on lifespan but also on functional healthspan, especially skeletal muscle function. If a longevity drug weakens muscle adaptation, it may help people live longer but make it harder for them to stay independent and mobile, which are essential for healthy aging. This paradox reflects a broader tension in geroscience: interventions that suppress anabolic signaling to extend lifespan may simultaneously impair tissue repair and regeneration [52]. Because skeletal muscle relies heavily on mechanical loading and anabolic signaling, these trade-offs are particularly important. The effects of Metformin on muscle are therefore unlikely to be uniformly “anti-aging” and instead appear to depend on context, acting primarily as a modulator of cellular energy sensing.

## 6. Conclusions and Perspective

**Figure 1:**
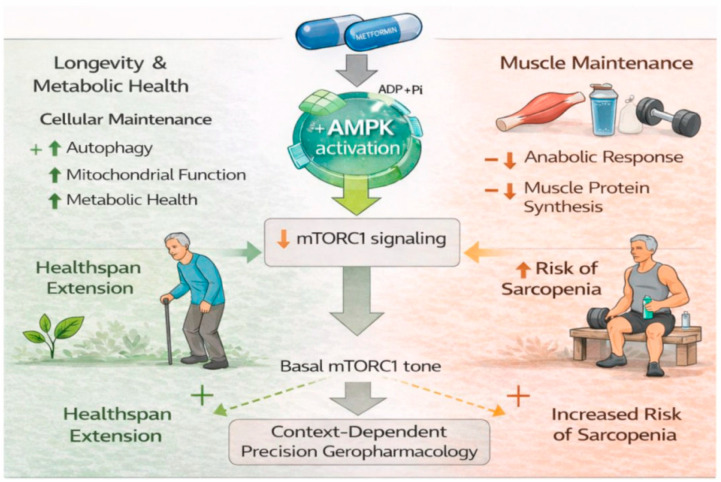
Schematic illustration of the context-dependent effects of metformin on longevity and sarcopenia. Metformin activates AMP-activated protein kinase (AMPK), promoting metabolic health, mitochondrial function, autophagy, and anti-inflammatory signaling that support longevity, particularly in frail or metabolically compromised older individuals. However, sustained AMPK activation concurrently suppresses mTORC1 signaling, a key driver of muscle protein synthesis and hypertrophic adaptation, potentially blunting resistance training–induced muscle growth and exacerbating sarcopenia in physically active older adults. These findings highlight a therapeutic paradox and emphasize the need for precision geropharmacological strategies in aging.

Taken together, the dual role of metformin as both a metabolic protector and a potential inhibitor of muscle adaptation challenges the notion of universal geroprotection (Figure 1). Consequently, the future of sarcopenia research lies in precision geropharmacology, in which interventions are tailored according to metabolic phenotype, exercise context, gut microbiome configuration, and individual aging trajectories. Rather than asking whether metformin is “good or bad” for aging muscle, more relevant questions are: when, in whom, at what dose, and under which metabolism and gut microbiome context does metformin confer benefit or harm? Hence, future studies should explicitly incorporate microbiome profiling into experimental and clinical designs, alongside integrated multi-omics, imaging, and functional endpoints, to delineate the dynamic drug–exercise–muscle axis. Only by embracing this biological complexity can metformin be transformed from a blunt anti-aging intervention into a refined and personalized strategy for preserving skeletal muscle health across the lifespan.

## Figures and Tables

**Table 1:** Representative findings illustrating the heterogeneous effects of metformin in aging-related diseases.

Year	Study	Model	Disease	Dose & Duration	Effect	Mechanism	Main Finding	PMID
2020	Meta-analysis	/	AD/PD	NA	Neg	/	No clear overall benefit for neurodegenerative diseases; possible increased PD risk.	32719079 [16]
2021	Clinical	AMD study	AMD in patients with diabetic retinopathy	/	Neg	/	No protection overall; increased risk in diabetics with retinopathy	33475696 [9]
2022	Meta-analysis	/	Cognitive impairment/ dementia-related outcomes	NA *	Pos	/	Association with reduced cognitive impairment risk	36090264 [11]
2022	Meta-analysis	/	age-related dementia	NA *	Pos	/	Association with a reduced risk of dementia subtype	35847477 [17]
2022	Meta-analysis	/	Cognitive impairment	NA *	Neg	/	No significant cognitive benefit; inconsistent evidence for dementia protection.	35297284 [12]
2023	in vivo	Tau-seeded PS19 mice	Alzheimer’s disease/ tauopathy	4 mg/mL p.o./4 months	Pos	↓ Tau hyperphosphorylation via ↓ mTORC1	Reduced tau pathology and improved cognition via mTORC1 downregulation.	36422837 [13]
2023	Clinical	/	Cognitive decline/ dementia-related pathology	/	Pos	/	Slower global cognitive decline in metformin users; Postmortem pathology assessed.	37931533 [10]
2024	in vivo	NT mice	AD	300 mg/kg/day p.o. 2 years,	Mixed	AMPKα1, Aβ plaques/oligomers, p-tau, GSK3β	Age- and model-dependent effects: cognitive benefit in young mice but worsened cognition and AD pathology in aged 3xTg-AD mice.	38238285 [14]
		3xTg-AD mice		300 mg/kg/day p.o. 1 year				
2025	in vitro	Senescent human fibroblasts	Skin aging/ impaired wound repair	2.5, 5, and 10 mM 72 h	Pos	SIRT1, FAP-α; senescence-associated dysfunction	Enhanced wound-healing behaviors in senescent fibroblasts, supporting anti-aging effects.	41196372 [18]
2025	ex vivo/ in vitro	Senescent B cells from fat in obese subjects	Immune aging/ inflammaging (obesity-linked immune senescence)	1 mM 24 and 48 h	Pos	β-gal; SASP/metabolic inflammatory signatures	Reduced B-cell senescence and SASP-like inflammation in vitro.	39389182 [15]

Abbreviation: Pos, positive effect; Neg, negative effect; Mixed, both positive and negative effect; AD, Alzheimer’s disease; PD, Parkinson’s disease; AMD, age-related macular degeneration; SASP, senescence-associated secretory phenotype; ↓, decreased. * NA (pooled exposure as defined in included studies).

**Table 2:** Reported mechanisms underlying the effects of metformin on sarcopenia (2020–2025).

Year	Study	Model	Dose/Period	Effect	Mechanism *	Main Findings	PMID
2020	in vivo	Grx1 knockout mice with spontaneous muscle atrophy; short-term MET	200 mg/kg/day i.p./15 days	Pos	AMPK/Sirt1; intramuscular lipid deposition; glucose utilization	Partial improvement of muscle atrophy in Grx1^−/−^ mice.	33069361 [33]
2020	clinical	Cross-sectional study	/	Pos	/	Lower sarcopenia risk	31914078 [29]
2021	Meta-analysis	Systematic review & meta-analysis (observational studies in T2D): prevalence/risk factors of sarcopenia	/	Pos	/	Sarcopenia is common in T2D; Lower risk of sarcopenia	34479652 [34]
2021	Meta-analysis	Network meta-analysis of RCTs (FFM as muscle-mass proxy)	/	NS	/	No significant effect on FFM vs. placebo	32628589 [30]
2021	in vitro/ex vivo	Human older-adult exposure (2-week metformin ingestion) with muscle progenitor cells studied in vitro	500 mg b.i.d. for the first 4 days, 1.5 g/day for the next 5 days, and 2 g/day for the final 5 days (Total 14 days)	Neg	Chromatin/histone +inflammation signaling	Mixed effects on aged MPCs: functional gains but enhanced inflammatory signaling.	33406027 [31]
2021	in vivo	Aging + disuse/recovery model; MET + leucine	336.6 mg/kg/d p.o./14~28 days	Pos	Satellite cell-related markers; fibrosis/collagen; muscle quality	Improved muscle function and quality during aging-related disuse/recovery.	34416035 [35]
2022	in vivo	Sarcopenic mouse models (also obese-sarcopenic) with 5-month metformin	200 mg/kg/day i.p./5 months	Pos	p-AMPKα (Thr172); inflammation; ectopic lipid; lipolysis (HSL/ATGL)	Improved muscle mass and function in sarcopenic mice; attenuated effects with obesity.	35905940 [36]
2022	clinical	Randomized controlled trial in healthy older adults (short-term metformin)	500 mg b.i.d. for the first 4 days, 1.5 g/day for the next 5 days, and 2 g/day for the final 5 days (Total 14 days)	Neg	Mitochondrial redox: ↑ mitochondrial H_2_O_2_ emission/production	Short-term ↑ mitochondrial H_2_O_2_ in muscle; potentially adverse for aging.	35405248 [32]
2023	in vitro	C2C12 Myotube atrophy models	MET (0.1μM) + LEU (0.5 μM)/single dose	Pos	Senescence/inflammation transcripts, proteostasis, AMPK-related signaling	Together with leucine reversing atrophy programs and preserving myotube size.	36947713 [37]
2023	in vivo	*C. elegans*	25 mM MET + 100 μM galantamine/29 days	Pos	Autophagy/lysosome; mitochondrial quality; satellite cells	Improved muscle function and quality via mitochondrial and autophagy pathways.	37551712 [27]
		Opa1^−/−^ mice	410 mg/kg/day MET + 3.28 mg/kg/day galantamine with Chow diet/90 days				
		Aged WT mice	410 mg/kg/day MET + 3.28 mg/kg/day galantamine with Chow diet/12~18 wks				
2024	in vitro	Late-passage C2C12 myoblasts (cellular aging/senescence model)	75 or 500 μM MET/once per 24 h	Pos	AMPK-linked improvement of autophagic flux and mitochondrial function	Reduced myoblast senescence and restored myogenesis via mitochondrial and autophagy improvements.	39533541 [28]

Abbreviation: Pos., positive effect; Neg., negative effect; NS, not significant; MET, metformin; FFM, fat-free mass; MPCs, muscle progenitor cells; ↑, increased; HSL, hormone-sensitive lipase; ATGL, adipose triglyceride lipase. NA, not applicable (e.g., observational/meta-analytic studies where dosing is not defined). * Reported mechanisms are based on associations or inferred pathways unless otherwise specified.

## Data Availability

No new data were created or analyzed in this study.

## Abbreviations

The following abbreviations are used in this manuscript:

AD	Alzheimer’s disease
AMPK	AMP-activated protein kinase
AMD	Age-related macular degeneration
ATGL	Adipose triglyceride lipase
CRP	C-reactive protein
FFM	Fat-free mass
FoxO3a	Forkhead box O3a
GSK3β	Glycogen synthase kinase-3 beta
HSL	Hormone-sensitive lipase
MASTERS	Metformin to augment strength training effective response in seniors
MET	Metformin
mTORC1	Mammalian target of rapamycin complex 1
MPCs	Muscle progenitor cells
MuRF1	Muscle RING finger protein-1
NA	Not applicable
NS	Not significant
PD	Parkinson’s disease
Pos	Positive effect
Neg	Negative effect
SASP	Senescence-associated secretory phenotype
SCFA	Short-chain fatty acid
T2DM	Type 2 diabetes mellitus
TAME	Targeting aging with metformin
